# Troponin Mutation Caused Diastolic Dysfunction and Experimental Treatment in Transgenic Mice with Cardiomyopathy

**DOI:** 10.7603/s40782-014-0017-6

**Published:** 2015-07-09

**Authors:** Yang Xu, Jie Tian, Xupei Huang

**Affiliations:** 1 pediatrics research institute in Children’s hospital, Chongqing Medical University, Chongqing, China; 2 Children’s Hospital, Chongqing Medical University, Chongqing, China

**Keywords:** *myofilament*, *gene mutation*, *cardiomyopathy*, *heart failure*, *transgenic mice*

## Abstract

Troponin, a contractile protein of the thin filament of striated muscle, consists of three subunits: troponin C (TnC), troponin T (TnT), and troponin I (TnI). Cardiac troponin I (cTnI) plays a critical role in regulation of cardiac function. The physiological effect of cTnI, as an inhibitory subunit of troponin complex, is to prevent the interaction between myosin heavy chain heads and actins, i.e. the cross-bridge formation, and to ensure a proper relaxation of cardiac myofilaments. In pathological conditions, the deficiency of cTnI or mutations in cTnI especially in the C-terminus of cTnI is associated with diastolic dysfunction caused by myofibril hypersensitivity to Ca^2+^. Our laboratory has generated cTnI knockout mouse model to investigate the cellular and molecular function of cTnI and created cTnI mutant disease mouse models to explore the pathophysiology caused by cTnI mutations in the heart. Here, we present our recent studies on physiological function of cTnI in the heart and the pathological consequences caused by the cTnI mutations in the diseased heart using the transgenic mouse models. The mechanisms underlying diastolic dysfunction and heart failure caused by cTnI mutations are explored in cell-based assays and in transgenic animal models. These studies provide us with useful information in searching for therapeutic strategies and target-oriented medication for the treatment of diastolic dysfunction and heart failure.

## I. Introduction

Heart failure is a leading cause of mortality in the United States. Up to 40% of heart failure patients have impaired relaxation, known as diastolic dysfunction and diastolic heart failure with normal systolic performance. Diastolic heart failure is an important clinical syndrome and both hypertrophic cardiomyopathy (HCM) and restrictive cardiomyopathy (RCM) share the same feature of diastolic dysfunction. It is known that single cardiac myofibril gene mutations are associated with cardiomyopathies leading to diastolic heart failures. However, the mechanisms underlying the impaired relaxation in myocardial cells and diastolic dysfunction developed in the heart are unknown. There is a critical need to understand the mechanisms that cause diastolic dysfunction and heart failure and to develop target-based intervention and medications to correct the overt condition and to treat the patients. For the moment, in the absence of such agents and intervention, treatment of diastolic dysfunction is difficult and often ineffective [1]. Our laboratory is among the first in studying the molecular mechanisms and pathophysiology of diastolic dysfunction and diastolic heart failure in transgenic mouse models. We have generated cardiac troponin I (cTnI) gene knockout mouse model [2] and transgenic mice with cTnI C-terminal mutations [3, 4]. Using these animal models, we have explored the physiological roles of troponin in cardiac muscle movement and cardiac function and revealed the mechanisms underlying cardiac dysfunction and heart disorders caused by the troponin mutations in myocardial cells.

## II. Materials and methods

### A. Generate cTnI knockout mouse model

Homologous recombination in embryonic stem (ES) cells was used to delete the entire cTnI gene from the mouse genome. In cTnI gene knockout mice (cTnI-KO), all homozygous mutants die on days 17 or 18 after birth, whereas the heterozygous mutants have a similar life span as that of the wild type mice [2]. By crossing two heterozygous mutants, we receive homozygous, heterozygous and wild type offspring for our studies.

### B. Create transgenic mice with RCM cTnI mutations

Our laboratory has created transgenic (TG) mice containing the RCM cTnI R193H and K178E mutations reported by Mogensen [5] and transgenic RCM mice containing the mutation R205H in the mouse genome corresponding to cTnI R204H mutation discovered in human RCM patients [6, 7]. The transgenic mice carrying RCM cTnI R193H mutation (cTnI^193His^) had 20-40% mortality rates and developed RCM phenotype similar to that observed in RCM patients [3]. However, cTnI^178Glu^ and cTnI^205His^ mice were lethal and died a couple of days after birth. By crossing the cTnI^193His^ mice with heterozygous cTnI-KO mice, we generated various transgenic mouse lines expressing different levels of cTnI R193H in myocardial cells [8].

### C. Monitor cardiac fucntion in vivo

We have analyzed the dose-dependent negative effect of cTnI^193His^ in RCM TG mice. The mortality rate was monitored and recorded in the different transgenic lines and their cardiac function was measured to determine any functional change related to the expression level of cTnI^193His^. Cardiac function assessments were performed by using a Vevo 770 High-resolution *In Vivo* Imaging System from VisualSonics (Toronto, ON, Canada). 10 mice (5 males and 5 females) per line including wild type were analyzed. The animals were first anesthetized with isoflurane at a concentration of 5% and then maintained at 1.1% isoflurane by a facemask during the procedure. Mouse body temperature and ECG measurement were taken during the whole process. The ventricular chamber sizes during systole and diastole, and the septal and ventricular wall thickness were measured through M mode imaging. B mode imaging 4-chamber view was taken to measure atrial size. The mitral inflow was evaluated with the Pulse-Wave Doppler and the following parameters measured: early ventricular filling (E wave); late ventricular filling caused by atrial contraction (A wave); ejection time (ET); isovolumetric relaxation time (IVRT); deceleration time (DT); isovolumetric contraction time (IVCT). Doppler Tissue Imaging (DTI) analyses were also performed to determine the mitral annulus velocity in the hearts. The E/A, E’/A’ and E/E’ ratios were calculated as reported previously [3, 8-10].

### D. Cell-based assays

Myocardial cells were isolated freshly from experimental mouse hearts. The following cell-based assays were performed on these myocardial cells.

#### 1) Determination of the sarcomere length and myofibril conformation.

The resting sarcomere length in both WT and RCM myocardial cells was measured using the IonOptix system (IonOptix Co., Milton, MA) as reported previously [9, 10]. Cardiac myocytes were isolated with a Langendorff perfusion cell isolation system (Cellutron) using the manufacturer’s protocol and cultured in mouse myocyte culture medium provided by the same company. An Olympus IX 71 microscope was used to visualize the cells. The myocardial cells isolated from the different lines of RCM TG and WT mouse hearts were field stimulated at 37º C, at 8 volts and frequencies varying from 0.2, 0.5, 1.0, to 2.0 Hz. At each frequency, a minimum of 12 contractions were used for the myocyte to achieve a steady state and 10 subsequent contractions were recorded for analysis. Cardiac myocytes’ sarcomere length at end diastolic stage for the different transgenic lines was measured and compared to that of WT cells.

#### 2) Determine the calcium dynamics in myocardial cells.

The calcium transients on the cardiomyocytes isolated from the various RCM transgenic lines and the WT mice were measured as reported previously [8, 10]. Freshly isolated cardiomyocytes were loaded with 2 μM of fura-2 AM (Molecular Probes) for 20 minutes at room temperature in Tyrode’s solution containing (in mM): 131 NaCl, 4 KCl, 1 CaCl_2_, 1 MgCl_2_, 10 Glucose, 10 HEPES, at pH 7.4 with 1% BSA and 0.02% pluronic F-127 (Molecular Probes). After 30 minutes of de-esterification of the fura-2 AM, the cells were bathed in Tyrode solution. For measurement, the fura-2 loaded cardiomyocytes were excited by UV light (360 and 380 nm, alternately) and the fura-2 emission wavelength (510 nm) was recorded with a photomultiplier tube. The kinetics of Ca^2+^ transients were analyzed in conjunction with myocyte mechanical measurements.

## III. Results

### A. Phenotype of cTnI deficiency in cTnI-KO mice

Two main TnI genes are expressed in the mammalian heart under the control of a developmentally regulated program. Fetal TnI, which is identical to slow skeletal TnI (ssTnI), is expressed first and predominates throughout embryonic and fetal development. Around embryonic day 10, cTnI begins to be expressed. Soon after birth, cTnI accounts for roughly half of the total thin-filament TnI content, and then cTnI predominates throughout adulthood in most animals, including humans. In the cTnI-KO mice, ssTnI was found to compensate for the absence of cTnI, but only temporarily, as ssTnI was eventually down-regulated in the cTnI-KO mouse heart.

Since ssTnI could be down-regulated as usual during the TnI isoform switch, a mammalian model with a myocardial TnI deficiency was created in homozygous mutants about two weeks after birth and all these homozygous mutant mice died on day 18 after birth when the TnI content was declined below 40% of the total TnI amount in the heart [2]. The histological and functional measurement results clearly showed that TnI depletion altered both resting and active mechanical properties of ventricular myocytes and caused a lethal phenotype with shortened sarcomere length and a restricted ventricle due to a severe diastolic dysfunction [2, 11]. The results also showed for the first time that expression of the adult cTnI isoform is not an essential component of the switch that terminates expression of the fetal ssTnI expression in the developing heart. This mouse strain with its predictable time-dependent loss of TnI is useful in our later investigations to establish the effects of TnI depletion on cardiac function *in vivo*.

### B. Phenotype of cTnI mutations in RCM cTnI^193His^ mice

Our laboratory has been among the first to begin to define the effect of the RCM cTnI mutation on the development of diastolic dysfunction. We have created transgenic mice (cTnI^193His^) expressing human RCM mutation cTnI R192H (cTnI R193H in mouse sequence) in the heart. The cTnI^193His^ mice do not present with significant hypertrophy or ventricular dilation. They have a phenotype similar to that in human RCM patients carrying the same mutation characterized morphologically by enlarged atria and restricted ventricles and functionally by diastolic dysfunction and diastolic heart failure [3]. We have demonstrated that impaired relaxation is a main manifestation in the RCM cTnI transgenic mice [9].

### C. Cellular and molecular mechanisms of RCM cTnI mutations

The results from the cell-based assays demonstrated that impaired relaxation was a key that causes restricted ventricle in the RCM cTnI R193H mice [9]. Calcium pCa curve measurement data indicated a significant curve left-shift in myofibers from the RCM cTnI^193His^ mice, indicating an increase of myofibril sensitivity to Ca^2+^ [10]. The data obtained from Ca^2+^ transient measurements and biochemical assays indicated that cTnI mutation caused myofibril Ca^2+^ hypersensitivity was a key factor resulting in a delayed calcium drop-off from the myofilaments and a delayed relaxation time [10, 12].

### D. Experimental animal assays

Based on our studies, we have demonstrated that Ca^2+^ hypersensitivity in myocardial cells caused by RCM cTnI mutations is a key that results in a delayed Ca^2+^ drop-off rate and an impaired relaxation. To confirm this, we have carried out experimental animal assays to desensitize the hypersensitivity in the RCM myocardial cells.

#### 1) Correct of Ca^2+^ hypersensitivity by cTnI-ND in RCM cTnI^193His^ mice

Proteolysis of the N terminal of cTnI (cTnI-ND) occurs as a functional adaptation in simulated microgravity and also under physiological and stress conditions [13]. The phosphorylable serine residues 23 and 24 are eliminated because of the truncation and the interaction of cTnI with TnC altered. The myofilament Ca^2+^ sensitivity was decreased in the presence of cTnI-ND and diastolic function improved. The therapeutic effect of this cTnI desensitizing molecule was investigated in our RCM cTnI^193His^ mice presenting damaged diastolic function. Double transgenic mice (Double-TG) were created that express both cTnI-ND and the cTnI R193H in the heart. The results indicated that Ca^2+^ sensitivity was restored in the animals accompanied with improved diastolic function and increased life span [10]. Such findings confirm that calcium desensitization is a promising alternative to restore diastolic function in RCM hearts and highlight the need to further explore this therapeutic option.

#### 2) Reverse diastolic dysfunction by chemical Ca^2+^ desensitizer catechins in RCM cTnI^193His^ mice

There is a great need to develop or find small molecules and chemical Ca^2+^ desensitizers that can be used to alter myofibril sensitivity for Ca^2+^ and myofilament sliding kinetics. A great concern about biological agents is their non-toxicity and their good bioavailability but there are few Ca^2+^ desensitizers possessing such qualities. The catechin, (-)-epigallocatechin-3- gallate (EGCg) has been newly found to possess Ca^2+^ desensitizing abilities via its interaction with cTnC [14, 15]. This compound is the most abundant catechin in green tea and is credited for the numerous health benefits attributed to green tea consumption. EGCg desensitizes myofilament to Ca^2+^ by forming a ternary complex with the C-terminal domain of troponin C and the anchoring region of cTnI [15]. The affinity of TnC to Ca^2+^ is reduced as a result which facilitates cardiac relaxation.

**Figure 1.**
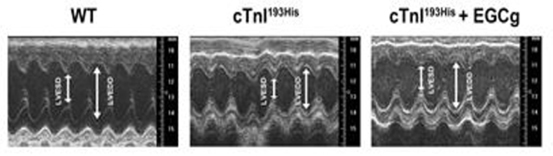
Decreased left ventricular end-diastolic dimension (LVEDD) was reversed in RCM cTnI^193His^ mice treated with EGCg.

**Figure 2.**
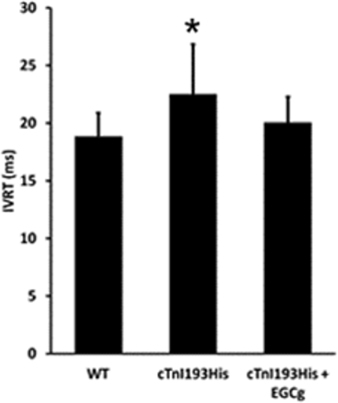
Prolonged isovolumetric relaxation time (IVRT) was corrected in RCM cTnI^193His^ mice treated with EGCg.

We have tested the therapeutic effects of the catechin EGCg in RCM mice. After daily intraperitoneal administration of 50 mg/kg body weight of EGCg over the course of 3 month, we observed in the echocardiography studies an improvement in the cardiac performance of the treated RCM cTnI^193His^ mice as shown by a restored left ventricle end diastolic dimension (LVEDD) in M mode imaging (Figure 1). In addition, the hemodynamic patterns of the RCM mice were improved after the treatment. Pulse Wave Doppler measurement of the mitral valve inflow in these animals showed a less prolonged isovolumetric relaxation time (IVRT) compared to untreated RCM cTnI^193His^ mice (Figure 2). The increased IVRT which is associated with impaired relaxation is the earliest manifestation of diastolic dysfunction in RCM animals. However, the long term effect of EGCg on the cardiac dysfunction and mortality rate still need to be studied to determine its full therapeutic potential for diastolic dysfunction and RCM.

## IV. Discussions

The contractile sarcomeric proteins consist of a highly ordered arrangement of myosin thick filaments, actin thin filaments, and associated proteins, such as the troponintropomyosin complex. Troponin, a contractile protein of the thin filament of striated muscle, consists of three subunits: troponin C (TnC), troponin T (TnT) and TnI. TnI is the inhibitory subunit which can bind to actin-tropomyosin and prevent muscle contraction by inhibition of actintropomyosin- activated myosin (actomyosin) ATPase activity. It has an important function in the regulation of striated muscle contraction. At basal levels of calcium, TnI inhibits actin-myosin crossbridges. In systole, as calcium binds to the regulatory site of TnC, the inhibitory action of TnI is released thereby activating muscle contraction. Cardiac contractionrelaxation is regulated mainly by intracellular calcium concentration and the proteins that are involved in the regulation of calcium cycling in myocardial cells, such as Ca^2+^ channel receptors, the SERCA2a calcium ATPase pump, phospholamban, etc. However, a body of studies has demonstrated that cTnI has unique functions in the control of cardiac muscle contraction and relaxation. In particular, the key role of cTnI in myocyte relaxation indicates a direct contribution of the myofilaments in modulating the dynamics of myocardial performance. For example, PKA-mediated cTnI phosphorylation causes a decrease of myofibril sensitivity to Ca^2+^ or a desensitization of the contractile apparatus to activation by Ca^2+^ and an acceleration cardiac myocyte relaxation performance.

Our studies have demonstrated that troponin is critical in the maintenance of a normal cardiac function and in the regulation of cardiac muscle movement. In the case of TnI deficiency or decrease, rest tension (Ca^2+^-independent force) is high in these myofibrils due to a tighter interaction between myosin and actin in the absence of TnI, which results in an impaired relaxation and diastolic heart failure even death. The fetal isoform of TnI, ssTnI, can compensate for the absence of cTnI by prolonging its expression time after birth. However, this compensation is temporary and the ssTnI gene turns off 18 days after birth in cTnI-KO mice. It is clinically important to understand the TnI isoform switching and the epigenetic regulation of TnI gene expression since the TnI content is reported to be decreased in aging hearts. If we understand clearly the regulation of TnI gene (cTnI or ssTnI), we can induce the expression of the TnI gene in case the heart needs more TnI.

From the studies using the transgenic RCM mice, we have demonstrated that hypersensitivity in RCM myocardial cells is a key factor that causes a delayed calcium drop-off rate from myofibrils and an impaired relaxation, which in turn results in diastolic dysfunction and heart failure. Our experimental therapeutic experiments have confirmed that desensitization is effective in correcting hypersensitivity of myofibril to Ca^2+^ and reversing the phenotype of RCM.

Our studies indicate that to have a normal cardiac contraction and relaxation, calcium concentration is critical. In addition, myofibril sensitivity to Ca^2+^ is important as well. It is clinically important to propose this novel concept since almost half of the heart failure patients have diastolic dysfunction and no effective medication is available so far for the diastolic dysfunction and heart failure. Calcium desensitization may be useful in the treatment of diastolic dysfunction and heart failure.
